# Evaluation of large language models in percutaneous coronary intervention decision-making

**DOI:** 10.3389/fcvm.2026.1690716

**Published:** 2026-04-02

**Authors:** Chengze Lin, Yongying Lan, Zi Zeng, Lingfang Zhuang, Rui Dai, Gang Lv, Yun Xie, Qi Jin, Liqun Wu, Qing Cao, Kang Chen

**Affiliations:** 1 Ruijin Hospital, Shanghai Jiao Tong University School of Medicine, Shanghai, China; 2 Shanghai Artificial Intelligence Laboratory, Shanghai, China

**Keywords:** clinical decision support, dynamic guidelines, large language models, model ensemble, percutaneous coronary intervention

## Abstract

**Background:**

Clinical decision-making for percutaneous coronary intervention (PCI) in patients with moderate-to-severe coronary stenosis is complex and sensitive to data completeness and guideline interpretation. We aimed to evaluate large language models (LLMs) for PCI support and to develop an ensemble framework for this complex decision setting.

**Methods:**

In this retrospective study, 15 LLM versions were evaluated using data of 93 patients from Ruijin Hospital. A hierarchical framework was employed to assess performance across varying data inputs. To optimize accuracy, advanced grouped ensemble strategies were developed and validated via nested repeated stratified 5-fold cross-validation. Probabilistic reliability and clinical utility were quantified through calibration plots and Decision Curve Analysis (DCA). Statistical robustness was ensured by bootstrap ROC-AUC comparisons with Holm-Bonferroni adjustment and restricted cubic spline modeling to analyze age-performance interactions.

**Results:**

Distinct behavioral patterns emerged across LLM families: Llama-3.3-70B-Instruct made more aggressive recommendations, whereas Grok-3 was more conservative. Holm-adjusted analysis identified significant performance gaps at age cut-points of 73, 75, and 76. A significant age-score interaction (LRT *p* = 0.00089) confirmed that patient age modulates model performance. The advanced ensemble strategies surpassed individual models, with an adaptive grouped ensemble achieving an F1 score of 0.921, compared to 0.807 for the best single model and 0.794 for a standard ensemble.

**Conclusion:**

Tailored LLM ensembles are feasible for PCI decision support and can improve robustness. Further multicenter prospective validation and multimodal integration are needed before clinical deployment.

## Graphical Abstract

**Figure:**
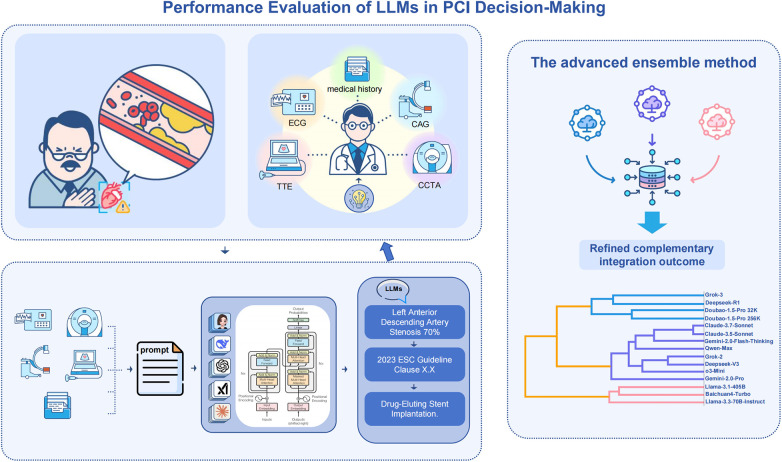

## 1 Introduction

Patients with coronary artery stenosis (diameter stenosis >50%–75%) face risks of angina, myocardial infarction, and sudden cardiac death, with coronary heart disease (CHD) ranking as the leading global cause of mortality (1). Percutaneous coronary intervention (PCI) as a key treatment for CHD can reduce mortality and improve prognosis (2). Currently, decision-making for PCI primarily relies on invasive coronary angiography and established clinical guidelines [e.g., the ESC Guidelines recommend FFR ≤0.80 as a decision threshold (3)]. However, the rapid updates of guidelines pose challenges even for experienced physicians in maintaining accuracy and consistency in patient data (4), Furthermore, due to the uneven distribution of medical resources, the quality and interpretation of coronary computed tomography angiography (CTA) reports exhibit significant heterogeneity across different regions,which,compounded by variations in medication use, patient transfers, and healthcare resource availability, increasing decision uncertainty and complicating the formulation of precise preoperative decisions from non-invasive data. In this context, intelligent optimization-driven approaches have demonstrated robust capacity for managing complex nonlinear parameter spaces in computational intelligence tasks, providing broader methodological grounding for AI-based clinical decision support (5).

In recent years, LLMs, pretrained on extensive natural language datasets, have demonstrated significant capabilities in contextual understanding, information extraction, and knowledge summarization, with recent advances in multi-step reasoning positioning them as candidate frameworks for clinical decision support. LLMs can generate transparent, traceable decision rationales, enabling clinicians to verify the evidence base underlying each recommendation and potentially improving adherence to guideline-concordant care (6). Furthermore, emerging evidence in cardiology suggests that beyond processing isolated imaging or text tasks, the access to clinical context substantially improves AI and LLM diagnostic accuracy. For instance, recent evaluations of GPT-4 in 12-lead electrocardiogram (ECG) interpretation have demonstrated that integrating detailed clinical narratives can mitigate the limitations of single-modality interpretation, although challenges in high-precision accuracy remain compared to experienced cardiologists (7, 8). Reinforcement learning from human feedback (RLHF) enables LLMs to align their outputs with expert preferences and evolving clinical guidelines (9), thereby offering a mechanism to standardize decision support across diverse practice settings while reducing biases arising from variations in individual clinicians’ experiential knowledge. Despite challenges in professional knowledge depth and output stability, LLMs’ interpretability and traceability establish an auditable framework for critical medical decisions (10), offering an efficient tool for shared physician-patient decisions.

This study examines PCI decisions for CHD patients, aiming to provide preliminaryevidence of LLMs’ feasibility in integrating current guidelines, extracting clinical evidence, and providing decision support. To introduce and evaluate a novel advanced ensemble methodology designed to overcome single-model limitations. Moreover, to address the impact of heterogeneous and noisy data inherent in clinical records, our ensemble framework is conceptually informed by the broader principle that intelligent algorithms can substantially enhance decision reliability in noise-prone environments (11). By situating LLMs within this robust framework, the study aims to clarify LLMs’ applicability and potential value in high-risk medical decision-making, delivering objective, timely decision-support tools to clinicians to improve decision accuracy, optimize healthcare resource allocation, and enhance reliability, and advance precision medicine.

## 2 Methods

### 2.1 Participants and task design

The participants in this study were 93 patients from Ruijin Hospital, Shanghai, enrolled between June 2015 and February 2025. The cohort had a mean age of 66.63 ± 9.30 years, with 67.7% being male. All participants were diagnosed with moderate-to-severe coronary artery stenosis (diameter stenosis >50%–75%) via Coronary Computed Tomography Angiography (CCTA).

To thoroughly evaluate the clinical decision performance of LLMs in determining the necessity of PCI, this study designed nine clinically relevant tasks (T1–T9), encompassing various input conditions and decision scenarios (Table 1). These tasks were developed to simulate clinical decisions under different information inputs and guideline constraints.

**Table 1:** Task design.

Task	Input	Guidelines	Design purpose
T1	Gender + Age + Surgery Time + CAG	Yes	Assess the model's accuracy in interpreting CAG-based parameters.
T2	Gender + Age + Surgery Time + CTA Report		Investigate the potential of CTA reports replacing CAG indecision-making.
T3	Gender + Age + Surgery Time + CTA + TTE + ECG		Evaluate the model's integration of multimodal diagnostic data.
T4	T1 + Chief Complaint + Past History + Present History		Assess the model's performance in integrating clinical history with diagnostic data.
T5	T2 + Chief Complaint + Past History + Present History		
T6	T3 + Chief Complaint + Past History + Present History		
T7	Gender + Age + Surgery Time + CAG	No	Measure the model's inference ability without guidelines.
T8	Gender + Age + Surgery Time + CTA Report		
T9	Gender + Age + Surgery Time + CTA + TTE + ECG		

The task design was divided into three groups. The first group (T1–T3) using angiography (CAG) reports (T1), CCTA reports (T2), and multimodal data [CCTA + echocardiography + electrocardiograms (ECG), T3] as inputs, respectively, requiring the models to adhere to guidelines. The second group (T4–T6) built upon the first group by adding chief complaints, present medical history, and past medical history. The third group (T7–T9) used the same inputs as the first group, but removed guideline constraints. The complete prompt text for all tasks is shown in Supplementary Figure S3.

### 2.2 Data collection

This retrospective study collected data from 93 patients diagnosed with moderate-to-severe coronary stenosis at Ruijin Hospital in Shanghai between June 2015 and February 2025. The inclusion criteria stipulated that patients must have had only a single coronary CAG per hospitalization or no additional PCI after their initial CAG. For each patient, a comprehensive set of clinical data was gathered, including text reports from CCTA, CAG, echocardiography, and ECG, in addition to chief complaints and medical histories. During data entry, only objective clinical findings and anatomical descriptions were extracted from reports; all treatment recommendations and decision-oriented content were excluded at the point of data collection (see Supplementary Materials for input examples). Then all collected data were subsequently standardized for consistency and anonymized prior to analysis to protect patient privacy.

### 2.3 Model selection

This study selected 15 versions of 9 distinct globally representative LLMs, including Claude-3.7-Sonnet, Grok-3, and Llama-3.3-70B-Instruct, among others. The release dates of the selected API versions are summarized in Table 2. With the exception of Grok-3, all models were tested by invoking API calls using default parameters to ensure result comparability; each query was input into an independent chat session to minimize bias (12).

**Table 2:** Release dates of selected API versions for models.

International models	API version release	Domestic models	API version release
Claude-3.5-Sonnet	2024-10-22	Baichuan4-Turbo	2024-05-22
Claude-3.7-Sonnet	2025-02-19	DeepSeek-R1	2025-01-20
Gemini-2.0-Flash-Thinking	2025-01-21	DeepSeek-V3	2024-12-26
Gemini-2.0-Pro	2025-02-05	Doubao-1.5-Pro-32k	2025-01-15
Grok-2	2024-12-12	Doubao-1.5-Pro-256k	2025-01-15
Llama-3.1-405B	2024-07-23	Qwen-Max	2025-01-25
Llama-3.3-70B-Instruct	2024-12-06		
O3-Mini	2025-01-31		

All versions of the LLaMA series models were accessed and tested via API provided by Google Cloud. Although Grok-3 had not made its API publicly accessible at the time of this study, it was still included in the evaluation due to its significant research value; all sample tasks for Grok-3 were manually tested through a web interface to maintain fairness and consistency in the evaluation process.

### 2.4 Study end point

The study endpoint was to compare the PCI necessity decisions recommended by LLMs with actual treatment outcomes, evaluating the models’ accuracy and practicality. In our clinical workflow, the actual treatment was not based on individual preference but followed a rigorous consensus-based protocol. Specifically, the ground truth was defined by the actual clinical intervention (PCI), which required a mandated, unanimous consensus among a three-tier physician team (comprising Chief, Associate Chief, and Attending Physicians) prior to execution. The subsequent decision for PCI was strictly guided by angiography results—the clinical reference standard—and performed in rigorous accordance with clinical guidelines. To ensure objective evaluation, models were strictly prompted for binary outputs (0/1).

### 2.5 Statistical analysis

#### 2.5.1 Global performance evaluation of models

To comprehensively evaluate the global performance, predictive consistency, and discriminative ability of the models, we first employed Principal Component Analysis (PCA) and t-distributed Stochastic Neighbor Embedding (t-SNE) to visualize the feature space, allowing for an intuitive assessment of the models’ ability to distinguish between True Positive (TP) and non-TP. Building on this, we calculated the predictive accuracy and consistency for each sample by each model across all tasks, Subsequently, we aggregated the performance of all models across all tasks,systematically calculating the average accuracy, sensitivity, specificity, F1 score, and Area Under the Receiver Operating Characteristic Curve (AUC-ROC) to summarize their overall performance. Finally, correlation analysis was used to quantify the degree of consistency in predictions among the 15 different models.

#### 2.5.2 Evaluation of specific tasks and single models

A more detailed evaluation was conducted at the level of specific tasks and individual models. We not only calculated the average performance metrics for all models on each individual task but also assessed intra-model stability for Tasks T4, T5, and T6, each case was queried three times per model; Fleiss’ kappa (κ) was employed to quantify within-model reproducibility (12). For individual models, we analyzed the distribution of their accuracy across all 9 clinical decision tasks to assess performance stability. Furthermore, we constructed a confusion matrix for each model's performance on each task, enabling a more detailed statistical analysis of performance metrics.

#### 2.5.3 Inter-Group performance comparison

To investigate the impact of different input information and constraints of instructions on model decision-making, we designed three groups of tasks for comparative analysis. The first comparison group (T1 vs. T4 vs. T7) aimed to evaluate performance differences among models under three scenarios: with guideline constraints based on CAG, with the addition of chief complaint and history of present illness, and without guideline constraints. The second group (T2 vs. T5 vs. T8) assessed the impact of adding chief complaint information and removing guideline constraints in tasks based on CCTA. The third group (T3 vs. T6 vs. T9), in a multimodal input setting, further analyzed the influence of incorporating additional medical history and removing guideline constraints on the models’ decision-making capabilities.

#### 2.5.4 Construction and evaluation of the ensemble voting mechanism

To meet the stringent precision demands of medical prediction tasks, and inspired by the principle that ensemble learning can enhance model performance (13), we designed and implemented an ensemble voting framework. This framework aims to optimize predictive performance and adapt to diverse clinical scenarios through task grouping and diversity-driven model selection.

To ensure the robustness and clinical applicability of our findings, we implemented an evaluation pipeline centered on nested repeated stratified 5-fold cross-validation, featuring an outer loop of 5 folds repeated 200 times and an inner loop of 3 folds to strictly isolate model selection and hyperparameter tuning from final performance evaluation, thereby eliminating optimism bias. Within the inner loop, we developed and compared three ensemble strategies: a standard ensemble benchmark selected from multiple candidate strategies (including top-N voting, majority voting, confidence-based, and weighted methods) via inner-loop cross-validation, a Global Advanced Ensemble that searches for a single robust combination across the entire dataset, and our proposed Advanced Grouped Ensemble which dynamically selects optimal model combinations for specific task clusters. For these advanced frameworks, a baseline model was first identified based on its F1-score to ensure high discriminative performance, while two additional models with maximum diagnostic complementarity were identified using a balance parameter β (grid-searched across {0.5, 1.0, 1.5, 2.0} to weigh individual performance against contribution to ensemble diversity. Furthermore, we conducted a fine-grained threshold search from 0.30 to 1.00 in steps of 0.05 to determine the optimal decision boundary tailored to each specific clinical task group. Given that each LLM was constrained to binary outputs (1 = PCI recommended, 0 = PCI not recommended), ensemble-stage scores were derived as weighted, normalized aggregates of constituent model predictions, effectively quantifying inter-model voting proportions. These voting scores were subsequently transformed into calibrated probabilities via Platt scaling or isotonic regression, with the optimal calibration strategy automatically selected through inner-loop cross-validation to facilitate threshold-based decision-making and probabilistic performance evaluation.

#### 2.5.5 Age stratification

Age-stratified subgroup analysis was conducted using bootstrap ROC-AUC comparisons across candidate cut-points (minimum *n* ≥ 10 per group) with Holm-Bonferroni adjustment (14, 15). For each candidate threshold, the AUC difference and corresponding two-sided *p*-values were calculated. To mitigate reliance on data-driven cut-points, we further utilized a logistic regression model where continuous age was modeled using a restricted cubic spline (df = 4). This allowed us to assess the interaction term between age and model scores, with overall significance determined via the LRT.

#### 2.5.6 Tools used

All data processing, analysis, and visualization were performed using Python (version 3.12.3). The study utilized the following open-source Python libraries to support various functional needs:Pandas (version 2.2.2): For data manipulation and management; NumPy (version 1.26.4): For numerical computations; Scikit-learn (version 1.4.2): For implementing machine learning algorithms and performance evaluation; SciPy (version 1.13.1): For advanced matrix operations and cluster analysis; Matplotlib (version 3.9.2) and Seaborn (version 0.13.2): For data visualization.

All code was executed in a reproducible environment and managed through version control to ensure transparency and repeatability. Analyses were conducted on a Linux system equipped with 256 GB of memory.

## 3 Results

This study included a total of 93 patients diagnosed with ‘moderate-to-severe coronary artery stenosis’ based on CCTA reports, with a mean age of 66.63 years (range 35–83 years). Males accounted for 67.7% and females 32.3%. All patients underwent CAG, with 68.1% ultimately receiving PCI and 31.9% not undergoing PCI.

### 3.1 Task group performance and model differences

**Figure 1:**
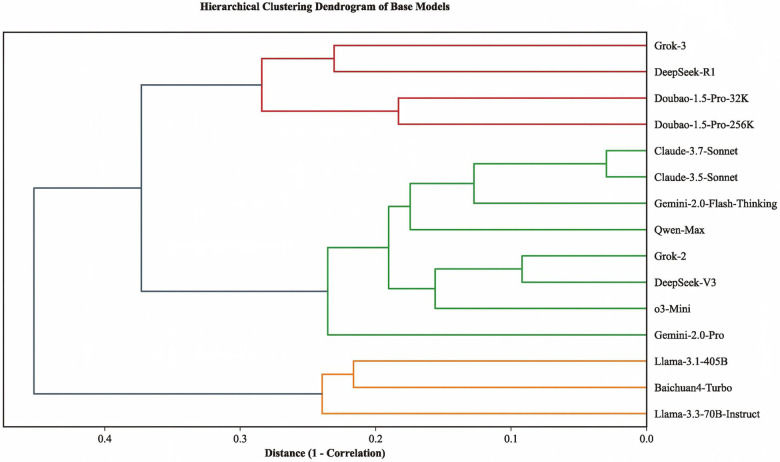
Hierarchical clustering of LLMs. This dendrogram illustrates the hierarchical clustering of 15 baseline models based on Spearman correlation coefficients. The horizontal axis represents distance, and the vertical axis lists model names. The models are grouped into three main clusters: (1) Grok-3, DeepSeek-R1, and Doubao-1.5-Pro (32K/256K); (2) Claude-3.7-Sonnet, Claude-3.5-Sonnet, and Gemini-2.0 series; and (3) Baichuan4-Turbo and Llama series.The clustering reflects differences in prediction styles among the models, providing a basis for model ensemble strategies.

**Figure 2:**
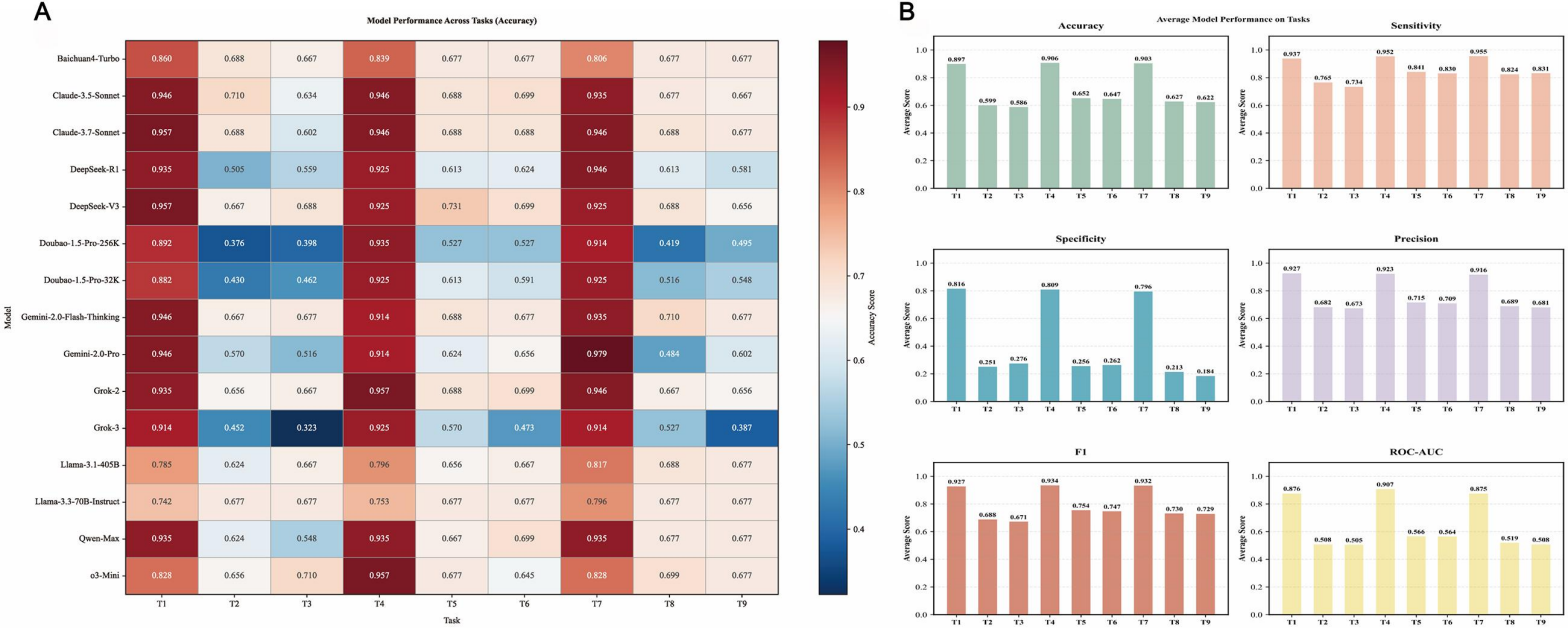
**(A)** Heatmap of accuracy for 15 models across tasks. This figure illustrates the accuracy performance of 15 large language models (LLMs) across nine clinical tasks (T1–T9). The horizontal axis represents the tasks, while the vertical axis lists the model names. The color intensity reflects the accuracy level, with darker shades indicating higher accuracy. **(B)** Line Chart of Average Performance of 15 Models Across All Tasks. This line chart depicts the average performance of 15 LLMs across all tasks (T1–T9), covering six metrics: Accuracy, Sensitivity, Specificity, Precision, F1 score, and ROC-AUC. The results highlight significant performance divergence among models across tasks, reflecting differences in their decision-making styles.

Claude-3.7-Sonnet exhibited the best performance across tasks T1 (CAG-based), T4 (CAG + chief complaints + medical history), and T7 (CAG, no guideline constraints), achieving accuracies of 95.7%, 94.6%, and 94.6%, respectively. Grok-3 had lower accuracies in T1–T3 (0.914, 0.452, 0.323) compared to T4–T6 (0.925, 0.570, 0.473), suggesting sensitivity to additional clinical information. Similarly, Doubao-1.5-Pro-32k showed accuracies of 0.882, 0.430, and 0.462 in T1–T3, rising to 0.925, 0.613, and 0.591 in T4–T6. Doubao-1.5-Pro-256k had accuracies of 0.892, 0.376, and 0.398 in T1–T3, increasing to 0.935, 0.527, and 0.527 in T4–T6, suggesting better performance in information-rich tasks compared to imaging-only tasks. In T7–T9 (CAG-based, no guideline constraints) vs. T1–T3 (CAG-based, guideline-required), removing guideline restrictions improved some models’ performance. Grok-3, with lower accuracies in T1–T3 (0.914, 0.452, 0.323), improved in T7–T9 (0.914, 0.527, 0.387). Doubao-1.5-Pro-32k's accuracies in T1–T3 (0.882, 0.430, 0.462) rose in T7–T9 (0.925, 0.516, 0.548). Doubao-1.5-Pro-256k's accuracies in T1-T3 (0.892, 0.376, 0.398) slightly improved in T7–T9 (0.914, 0.419, 0.405). DeepSeek-R1's accuracies in T1–T3 (0.935, 0.505, 0.559) increased in T7–T9 (0.946, 0.613, 0.581). These findings suggest that the evaluated models perform better in open-domain scenarios than when adhering to expert guidelines. We hypothesize that these models may share similar training data or architectural designs, favoring open-domain reasoning over strict guideline reliance. Figure 1 further reveals that DeepSeek-R1, Doubao-1.5-Pro-32k, Doubao-1.5-Pro-256k, and Grok-3 cluster together, indicating similarity in prediction styles (Figure 2A). Llama-3.3-70B-Instruct exhibited the highest sensitivity (99.6%), indicating its ability to effectively identify nearly all patients requiring PCI. Doubao-1.5-Pro-32k's high specificity (78.9%) demonstrated its excellence in ruling out patients not requiring PCI (Figure 2B).

### 3.2 Information integration capabilities and model characteristics

**Figure 3:**
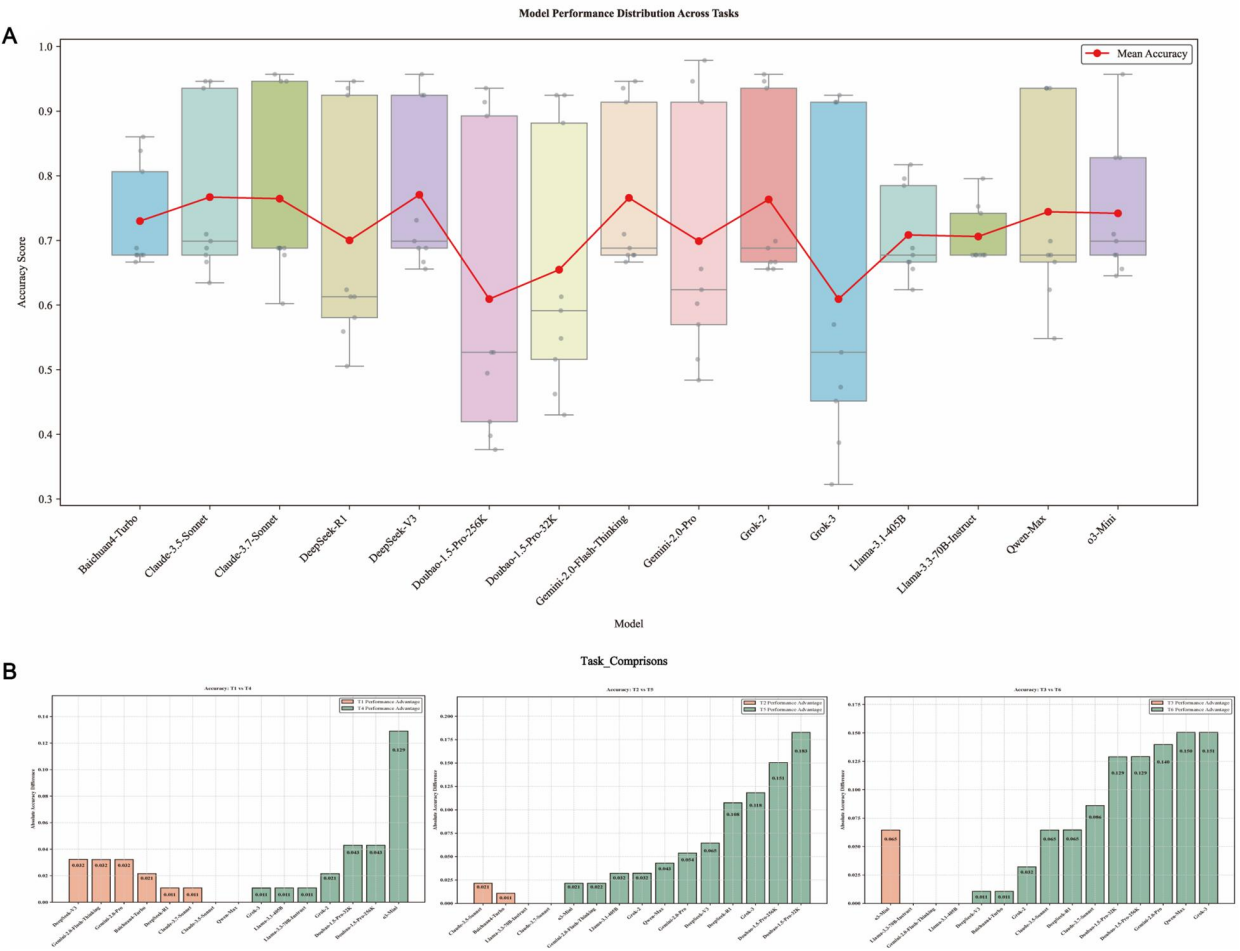
**(A)** Box plot of accuracy distribution across tasks for 15 models. This box plot presents the accuracy distribution of 15 models across all tasks, showing the median and interquartile range for each model's accuracy, with a line overlay indicating the mean accuracy. Grok-3 exhibited the widest accuracy range (0.323–0.925), suggesting it is heavily influenced by task inputs. In contrast, Llama-3.3-70B-Instruct demonstrated higher stability, with a more compact accuracy distribution. **(B)** Histogram of Accuracy Differences for 15 Models in T1 vs. T4, T2 vs. T5, and T3 vs. T6. This histogram illustrates the absolute accuracy differences of 15 models between tasks T1 vs. T4, T2 vs. T5, and T3 vs. T6 (orange: T1–T3 superior; green: T4–T6 superior). Grok-2, Grok-3, Doubao-1.5-Pro-32k, and Doubao-1.5-Pro-256k all performed better in T4, T5, and T6, with improved accuracy, demonstrating strong information integration capabilities.

Llama-3.3-70B-Instruct demonstrated the most stable performance, with a compact accuracy distribution, while Grok-3 showed the widest accuracy range (0.323–0.925), suggesting significant sensitivity to input variations. Although Grok-3 had lower accuracies in T2, T3, T5, T6, T8, and T9 and frequently recommended ‘Treatment Decision 0’ (no PCI), its outputs aligned more closely with guidelines, supported by comprehensive reasoning chains (Figure 3A). Grok-3, Grok-2, Doubao-1.5-Pro-32k, and Doubao-1.5-Pro-256k outperformed in T4–T6 compared to T1–T3, showing significant accuracy improvements (Figure 3B). These results suggest that Grok-3 and the Doubao series possess strong information integration capabilities, effectively processing complex inputs.

### 3.3 Model consistency

**Figure 4:**
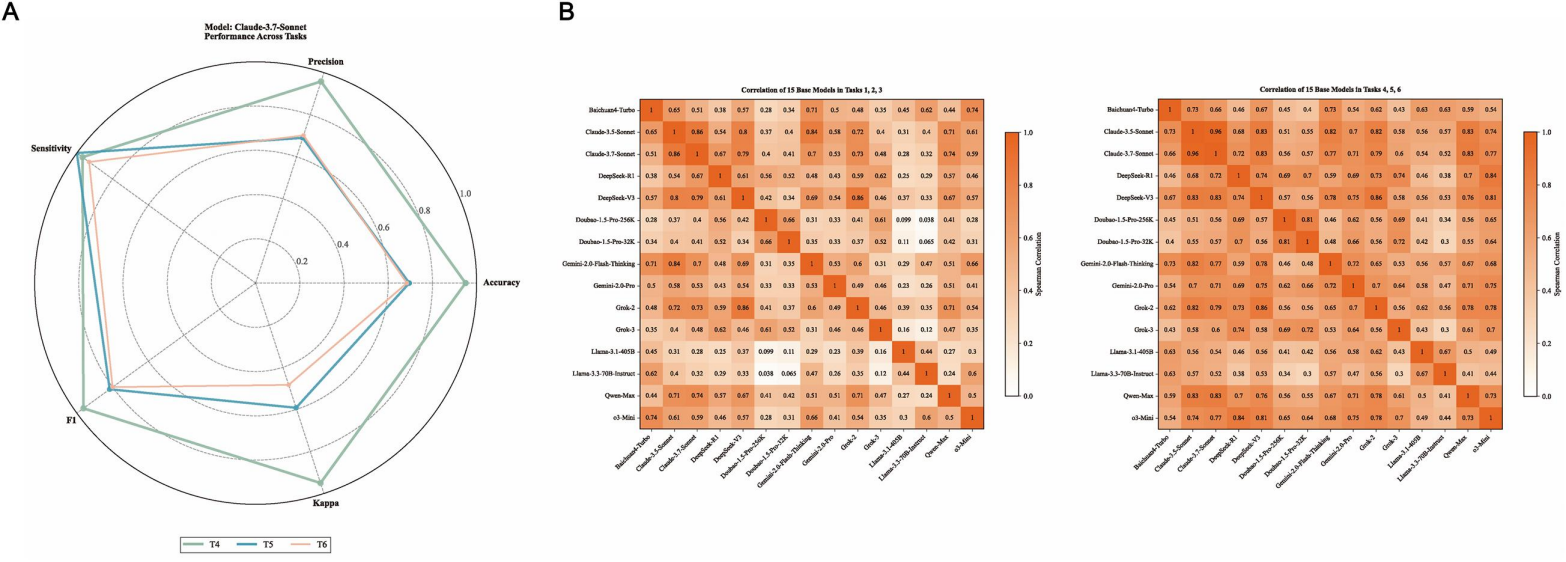
**(A)** radar chart of claude-3.7-Sonnet's Performance in Tasks T4, T5, and T6. This radar chart displays the performance of Claude-3.7-Sonnet in tasks T4 (green), T5 (blue), and T6 (orange), evaluated across six metrics: Accuracy, Sensitivity, Specificity, F1 score, Precision, and Consistency (Kappa). In T4, Claude-3.7-Sonnet achieved an accuracy of 0.95 and an F1 score of 0.97. Figure **(B)** Spearman Correlation Matrix of 15 Models in Tasks T1–T3 and T4–T6. This figure shows Spearman correlation matrices for the 15 models in tasks T1–T3 (left panel) and T4–T6 (right panel), with color intensity representing the magnitude of correlation coefficients. In T1–T3, the correlation between Claude-3.5-Sonnet and Claude-3.7-Sonnet was 0.86, increasing to 0.96 in T4–T6. Similarly, the correlation between Doubao-1.5-Pro-32k and Doubao-1.5-Pro-256k rose from 0.66 (T1–T3) to 0.81 (T4–T6). These results indicate that the addition of chief complaints and medical history significantly enhanced inter-model prediction consistency.

Within-model reliability analysis for Tasks T4–T6 demonstrated high intra-model stability. For instance, Claude-3.7-Sonnet achieved high reproducibility across repeated queries (Figure 4A); radar charts for other models are provided in the Supplementary materials. We further evaluated inter-model agreement to assess the alignment between different model versions. Claude-3.5 and Claude-3.7 exhibited higher consistency in T4–T6 (0.96) than in T1–T3 (0.86). Similarly, consistency of Doubao-1.5-Pro-32k and Doubao-1.5-Pro-256k improved from 0.66 in T1–T3 to 0.81 in T4–T6 (Figure 4B).

### 3.4 Subgroup analysis

**Figure 5:**
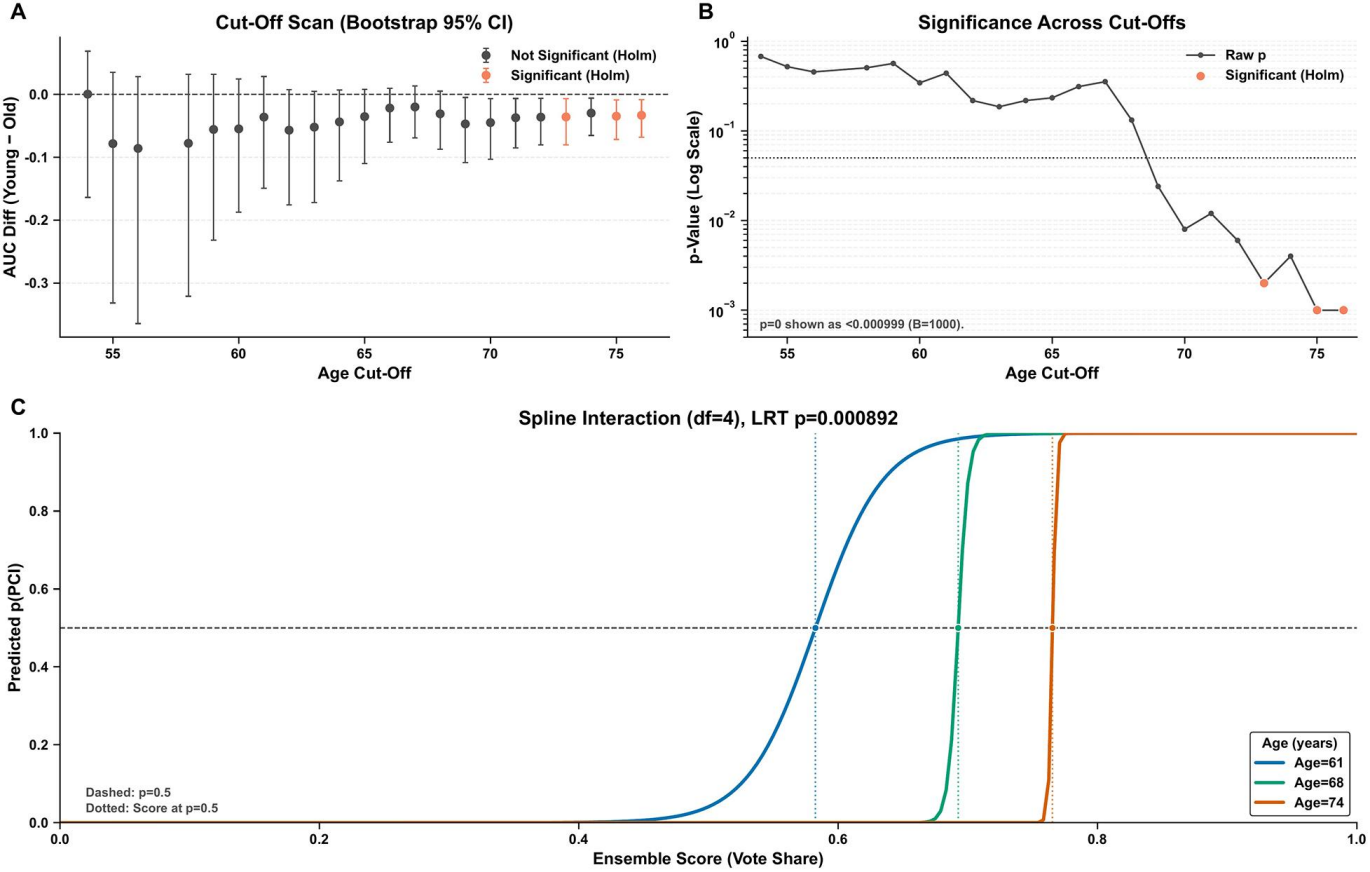
Analysis of model performance across the age spectrum. **(A)** Differences in area under the receiver operating characteristic curve (*Δ*AUC) between younger and older subgroups across varying age cut-offs, with 95% confidence intervals derived from 1,000 bootstrap replications. Statistically significant differences after Holm-Bonferroni correction are highlighted; the dashed horizontal line denotes null effect (*Δ*AUC = 0). **(B)** Corresponding two-sided bootstrap *p*-values (log10 scale) for each cut-off; *p* = 0 indicates *p* < 1/(B + 1). The horizontal reference line represents the significance threshold (*α* = 0.05). **(C)** Predicted probability of PCI as a function of ensemble score at the 25th, 50th, and 75th age percentiles, modeled using logistic regression with restricted cubic spline (4 degrees of freedom) interaction between continuous age and ensemble scores. Vertical dotted lines indicate score thresholds corresponding to 50% predicted probability for each age group. The significant age-by-score interaction (likelihood ratio test *p* = 0.00089) indicates that age modifies the discriminative performance of the model.

After Holm-Bonferroni adjustment, significant differences persisted only for cut-points 73, 75, and 76. Furthermore, the spline-based interaction analysis (LRT *p* = 0.00089) provided robust evidence that model performance varies non-linearly with age (Figure 5).

### 3.5 Voting mechanism experiment

Under the nested validation framework, the grouped advanced ensemble demonstrated superior performance compared to both single models and standard ensembles, achieving an F1-score of 0.921 ± 0.056 [95% CI: 0.783–1.000] and an accuracy of 0.893 ± 0.075 [95% CI: 0.722–1.000]. In the inner loop selection process, Claude-3.5-Sonnet was most frequently identified as the performance cornerstone, being selected in 77.9% of iterations. For the global ensemble, the most stable combination consisted of Gemini-2.0-Pro, Llama-3.3-70B-Instruct, and Doubao-1.5-Pro-32K, which appeared in 25.5% of the selection processes. This advanced ensemble configuration outperformed the best single model (F1: 0.807 ± 0.020) and the optimized standard ensemble (F1: 0.794 ± 0.053). Holm-adjusted pairwise tests confirmed that these improvements were statistically significant across F1 (*p* < 0.05, paired bootstrap) and Accuracy (*p* < 0.05, McNemar); details are presented in Table 3.

**Table 3:** Performance comparison between best single model and advanced ensemble.

Method	F1	Accuracy	ROC-AUC	Brier	ECE	MCE
Best single model	0.807 ± 0.020 [0.759, 0.839]	0.682 ± 0.031 [0.611, 0.737]	0.933 ± 0.066 [0.782, 1.000]	0.155 ± 0.024 [0.116, 0.210]	0.210 ± 0.028 [0.152, 0.275]	0.662 ± 0.111 [0.467, 0.889]
Standard ensemble	0.794 ± 0.053 [0.667, 0.880]	0.703 ± 0.066 [0.556, 0.833]	0.96 ± 0.036 [0.872, 1.000]	0.136 ± 0.017 [0.102, 0.172]	0.211 ± 0.031 [0.152, 0.269]	0.581 ± 0.111 [0.326, 0.793]
Advanced ensemble(global)	0.880 ± 0.064 [0.750, 1.000]	0.835 ± 0.086 [0.667, 1.000]	0.898 ± 0.080 [0.705, 1.000]	0.122 ± 0.057 [0.020, 0.243]	0.130 ± 0.062 [0.037, 0.268]	0.460 ± 0.209 [0.125, 0.859]
Advanced ensemble(group)	0.921 ± 0.056 [0.783, 1.000]	0.893 ± 0.075 [0.722, 1.000]	0.957 ± 0.049 [0.833, 1.000]	0.078 ± 0.040 [0.016, 0.173]	0.139 ± 0.042 [0.067, 0.230]	0.531 ± 0.176 [0.231, 0.892]

However, the best single model (primarily Claude-3.5-Sonnet) exhibited a remarkably low mean specificity of 0.049 (95% CI: 0.033–0.100; Supplementary Table S2). This indicates that while achieving high sensitivity, single high-performing models demonstrated a systematic “aggressive” bias, misclassifying nearly all true-negative patients in CCTA and multimodal tasks. By incorporating model diversity constraints and threshold optimization, the grouped advanced ensemble successfully restored the specificity to 0.825, effectively balancing the predictive profile for clinical utility.

Regarding probabilistic performance, the grouped advanced ensemble achieved a high degree of calibration with a mean Brier score of 0.078 ± 0.040 [95% CI: 0.016–0.173], which was superior to the advanced global ensemble [0.122 ± 0.057 (95% CI: 0.020–0.243)]. Calibration curves provided in Supplementary Figure S1 confirmed that the recalibrated ensemble outputs closely aligned with observed clinical outcomes, and exhibited lower ECE and MCE compared to baseline models.

The clinical value of the framework was further highlighted by Decision Curve Analysis in Supplementary Figure S2, which revealed that the grouped ensemble provided a higher clinical net benefit across a wide range of threshold probabilities, from 0.05 to 0.5, compared to the best single model. This evidence suggests that the ensemble framework, particularly when optimized through specific grouping, can effectively assist in PCI decision-making

## 4 Discussion

The selection of cases in this study adhered to strict and explicit clinical criteria, with the clinical reference standard for PCI indications established through the consensus of three cardiologists. We observed significant performance variations across task groups among different Large Language Models (LLMs). For instance, Grok-3 and Doubao models exhibited marked improvement in tasks integrating comprehensive clinical data (T4–T6) compared to basic information extraction tasks (T1–T3). This trend underscores their high sensitivity to clinical context and robust information integration capabilities. These findings align with recent machine learning studies in acute cardiovascular settings, which demonstrate that advanced algorithms can improve risk stratification and predictive accuracy by handling high-dimensional, non-linear clinical data—capabilities that are becoming increasingly vital in high-stakes care (16).

The performance of LLMs in PCI decision-making varies due to their distinct architectural designs and training strategies, impacting their clinical utility and consistency. Llama-3.3-70B-Instruct exhibited the highest sensitivity, favoring PCI recommendations and suiting rapid-decision scenarios, while Doubao-1.5-Pro-32k excelled in specificity, avoiding unnecessary interventions. Grok-3 exhibited notable performance fluctuations and high sensitivity to input changes, possibly reflecting its design emphasis on rapid interaction and dynamic processing (17). Gemini's multimodal architecture and ecosystem integration may enhance cross-modal reasoning (18). LLaMA's open-weight design with architectural optimizations (RMSNorm, SwiGLU, RoPE) trained on publicly available data offers a balance between efficiency and transparency (19). DeepSeek is based on deep neural networks,employing pruning and quantization techniques for model compression, with a focus on information retrieval (20, 21).These architectural differences—rooted in the Transformer core (22) - determine their effectiveness in high-precision medical contexts like PCI assessment. Clinically, aggressive models like Llama-3.3-70B-Instruct and conservative ones like Grok-3 suggest that model selection should consider patient conditions, treatment priorities, and local resources to optimize diagnostic efficiency and safety. Consistency analysis further revealed that Claude-3.5 and Claude-3.7 Doubao-1.5-Pro-32k and Doubao-1.5-Pro-256k achieved higher inter-model correlation in T4–T6 than T1–T3, with richer context from chief complaints and medical history enhancing agreement, suggesting shared architectural or developmental logic.

To address the limitations of individual models, we introduced a similarity-based grouping ensemble strategy. The conceptual design of this ensemble framework echoes the broader principles of intelligent optimization paradigms that address noise and uncertainty in complex identification problems (11). By synthesizing outputs from diverse model families, our approach effectively alleviates the noise and uncertainty inherent in clinical narratives, positioning our clinical application within the broader context of robust hybrid intelligent systems. Validated through nested cross-validation (Nested CV), this framework achieved a superior F1-score and a lower Brier score compared with the global ensemble, demonstrating high reliability in probability estimation. DCA further confirmed that the ensemble framework provided a higher clinical net benefit than the best-performing single models across a wide range of risk thresholds. Our results fit within a broader shift from purely statistical inference to machine-learning-augmented decision support in cardiovascular therapeutics, representing a paradigm shift toward more personalized and data-driven pharmacotherapy and intervention strategies (23). By integrating the complementary strengths of diverse model families, this approach effectively balances aggressive and conservative decision-making, optimizing both diagnostic efficiency and patient safety.

The low specificity of the best single model (mean 0.049; 95% CI: 0.033–0.100) has significant clinical implications. This aggressive recommendation bias leads to unnecessary procedural risks and medical resource waste in actual PCI practice. Such a phenomenon indicates that F1-score optimization often fails to penalize low specificity in imbalanced clinical datasets. In contrast, our advanced ensemble restored specificity to 0.825 while maintaining 0.925 sensitivity, demonstrating the necessity of model diversity and threshold optimization. We emphasize that future clinical LLM evaluations should report both sensitivity and specificity rather than relying solely on F1-scores to ensure patient safety and utility.

Despite these promising results, several limitations must be acknowledged. This study employed a three-cardiologist consensus as the reference standard; while clinically pragmatic, this approach may reflect operator preferences and institutional biases, rendering findings exploratory pending validation through physiological metrics (FFR/iFR) or blinded Heart-Team adjudication. To isolate intrinsic reasoning, we excluded established diagnoses and biomarkers—a design that clarifies LLM capabilities on unstructured data but may constrain accuracy, necessitating future assessment of incremental diagnostic value from incorporating these robust clinical indicators. Then, as a proof-of-concept exploratory study, the sample size of 93 patients from a single medical center (Ruijin Hospital) is a primary limitation. Given the lack of large-scale, public multicenter datasets for LLM decision support in cardiology, this study serves as a necessary exploratory initiative; however, future prospective, multicenter trials are required to validate generalizability. Furthermore, while this study confirmed the feasibility of binary decision-making for PCI indications, it did not address the identification of culprit coronary lesions—a cornerstone of precision intervention. Identifying culprit lesions requires refined spatial logic and deep anatomical understanding. Future research should therefore incorporate more detailed anatomical descriptions and raw imaging data to evaluate LLM performance in granular tasks such as culprit vessel localization. Additionally, the reliance on CCTA text reports may have constrained feature extraction. Future advancements should involve Multimodal Large Language Models (MLLMs) to directly process raw imaging data (24) and the integration of knowledge graphs to minimize hallucinations. Combining LLMs with explainable AI techniques will further enhance clinician trust and ensure alignment with the rigorous needs of precision medicine.

## Data Availability

The raw data supporting the conclusions of this article will be made available by the authors, without undue reservation.

## Supplementary material

Supplementary Figure S1 Calibration curves for the four evaluation methods. Calibration of the best single model, standard ensemble, advanced ensemble (global), and advanced ensemble (group) is assessed by comparing predicted probabilities (*x*-axis) against observed frequencies (*y*-axis). The diagonal dashed line represents perfect calibration (y = x). Reliability of probabilistic outputs was evaluated using a 10-bin strategy.

Supplementary Figure S2 Decision Curve Analysis (DCA) plot. Net benefit curves for the four modeling strategies are plotted across threshold probabilities (0.05–0.50) for PCI. The dashed line represents the “treat-all” strategy (assuming all patients undergo PCI). Net benefit quantifies the trade-off between true-positive and false-positive classifications at each decision threshold, with higher values indicating superior clinical utility.

Supplementary Figure S3 Unified prompt template used across nine clinical task groups (T1–T9). Color-coded superscripts mark task-specific elements, whereas elements without superscripts are shared across task groups.
