# Supplementary figures and images for: Evaluation of large language models in percutaneous coronary intervention decision-making
Source: Front Cardiovasc Med. 2026 Apr 2;13:1690716. doi: 10.3389/fcvm.2026.1690716 (PMC13083164; doi:10.3389/fcvm.2026.1690716)

# Calibration Plot

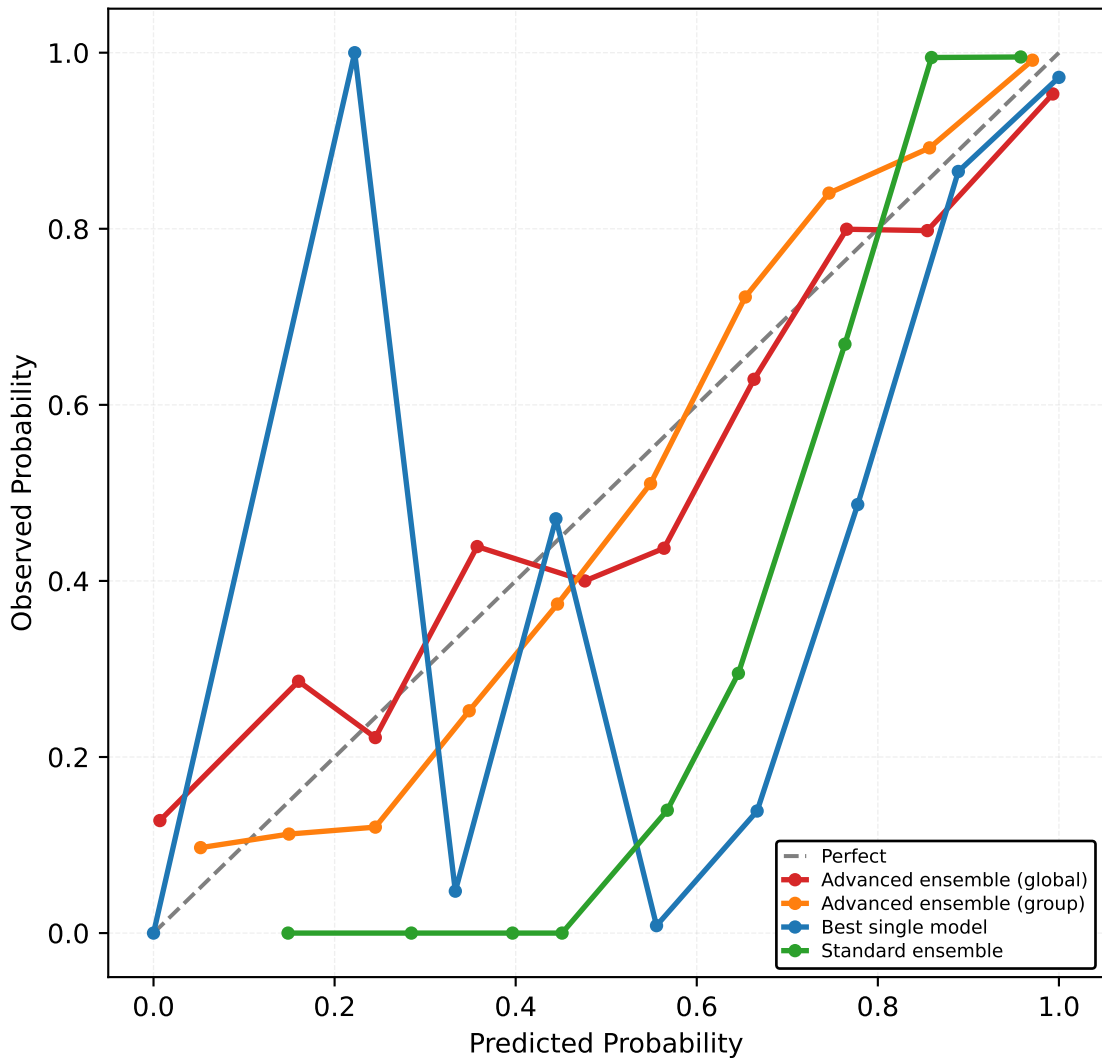

Supplement: Supplementary Figure S1 — Calibration curves for the four evaluation methods. Calibration of the best single model, standard ensemble, advanced ensemble (global), and advanced ensemble (group) is assessed by comparing predicted probabilities (x-axis) against observed frequencies (y-axis). The diagonal dashed line represents perfect calibration (y = x). Reliability of probabilistic outputs was evaluated using a 10-bin strategy. [file Image1.pdf]

## Decision Curve Analysis

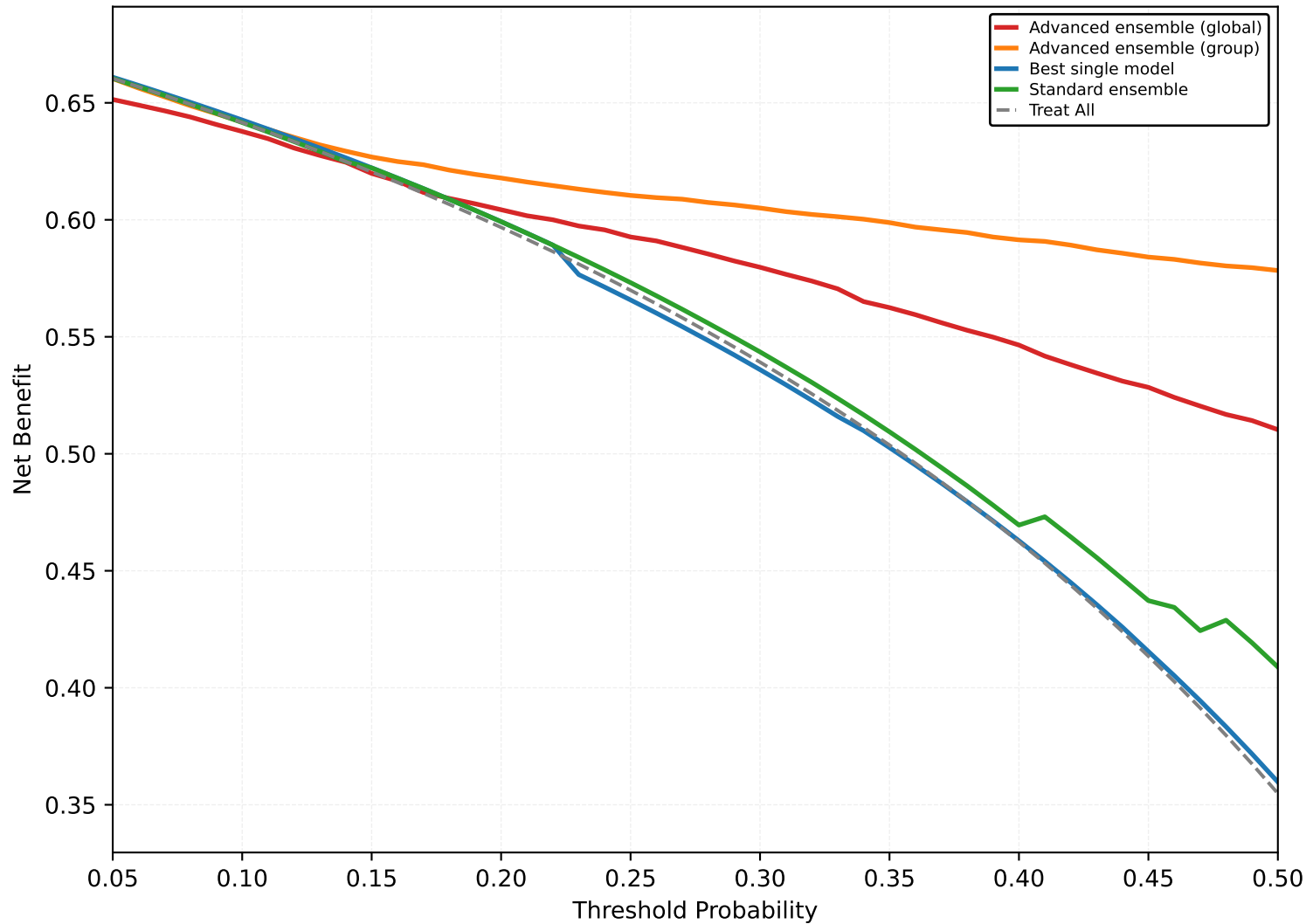

Supplement: Supplementary Figure S2 — Decision Curve Analysis (DCA) plot. Net benefit curves for the four modeling strategies are plotted across threshold probabilities (0.05–0.50) for PCI. The dashed line represents the “treat-all” strategy (assuming all patients undergo PCI). Net benefit quantifies the trade-off between true-positive and false-positive classifications at each decision threshold, with higher values indicating superior clinical utility. [file Image2.pdf]
