# Supplementary material for: Evaluation of large language models in percutaneous coronary intervention decision-making
Source: Front Cardiovasc Med. 2026 Apr 2;13:1690716. doi: 10.3389/fcvm.2026.1690716 (PMC13083164; doi:10.3389/fcvm.2026.1690716)
Supplement: Supplementary Figure S3 — Unified prompt template used across nine clinical task groups (T1–T9). Color-coded superscripts mark task-specific elements, whereas elements without superscripts are shared across task groups. [file Image3.pdf]

You are a cardiac intervention specialist. Based on the patient's clinical data and coronary [a1]angiography / [a2]CTA / [a3]multimodal imaging report, please determine whether PCI (Percutaneous Coronary Intervention) is needed according to [c]the latest cardiovascular intervention guidelines before the patient's surgery date, and recommend the best treatment plan. Please follow the process strictly:

[PATIENT INFORMATION]
Age: <age>{{AGE}}</age>
Gender: <gender>{{GENDER}}</gender>
[b]Chief Complaint: <chief complaint>{{CHIEF COMPLAINT}}</chief complaint>
[b]Present History: <present history>{{PRESENT HISTORY}}</present history>
[b]Past History: <past history>{{PAST HISTORY}}</past history>
Surgery Date: <day>{{DAY}}</day>
[a1]Coronary Angiography (CAG) Report:
<angiography>{{ANGIOGRAPHY_RESULTS}}</angiography>
[a2]Coronary CTA (CCTA) Report:
<CTA>{{CTA_RESULTS}}</CTA>
[a3]ECG Report:
<ECG>{{ECG_RESULTS}}</ECG>
[a3]Transthoracic Echocardiography (TTE) Report:
<TTE>{{TTE_RESULTS}}</TTE>

[STEP 1] Anatomical Feature Analysis (Required):
List in <analysis>:
* Lesion Location (Left Main / LAD / LCX / RCA etc.)
* Stenosis Degree (percentage)
* Lesion Type (A / B1 / B2 / C)
* Special Features (Calcification / Thrombus / Bifurcation etc.)

[STEP 2] Indication Assessment[c] (Must cite guidelines):
[c]Reference the latest cardiovascular intervention guidelines before surgery date:
ESC CCS Guidelines / ESC ACS Guidelines /
ACC/AHA/SCAI Coronary Revascularization Guideline /
Chinese PCI Guidelines
  * [c]Cite specific provisions (e.g., "ESC NSTE-ACS Guidelines Chapter X, Item Y")

Assess if meeting following indications:
* Acute Coronary Syndrome
* High-risk Chronic Coronary Syndrome
* Significant Ischemic Evidence
* Left Main Disease ≥50%
* Proximal LAD Stenosis ≥70% etc.

[STEP 3] Treatment Decision:
If PCI indicated, select procedure based on lesion characteristics (with reasoning):
* Balloon Dilation
* Drug-Eluting Stent Implantation
* Jailed-wire Balloon
* Cutting Balloon
* Drug-Coated Balloon

[REQUIRED OUTPUT FORMAT]
<decision>
Treatment Decision: [1 = PCI recommended / 0 = PCI not recommended]
</decision>
<basis>
[[c]Brief explanation citing specific guideline provisions]
</basis>
<recommendation>
Recommended Plan: [Specific procedure]
[c]Guideline Reference: [Guideline name] Chapter [X], Item [Y]
Reason: [Combined with lesion characteristics]
</recommendation>

---

a1 Applied in T1, T4, T7 only. CAG report provided as sole imaging input.

a2 Applied in T2, T5, T8 (CCTA alone) and T3, T6, T9 (CCTA as part of multimodal input).

a3 Applied in T3, T6, T9 only. ECG and TTE reports added alongside CCTA (multimodal input).

b Applied in T4, T5, T6 only. Chief Complaint, Present History, and Past History are omitted in all other tasks.

c Applied in T1–T6 only. In T7, T8, T9: (i) the role description replaces "the latest cardiovascular intervention guidelines" with "available clinical data"; (ii) the guideline citation instruction and citation format in Step 2 are removed; (iii) the "Guideline Reference" line in the output is omitted.

Tasks T4, T5, and T6 were each administered three times using identical prompts (denoted $T4_1/T4_2/T4_3$, $T5_1/T5_2/T5_3$, $T6_1/T6_2/T6_3$) to assess within-model response consistency.
