# Supplementary material for: Evaluation of large language models in percutaneous coronary intervention decision-making
Source: Front Cardiovasc Med. 2026 Apr 2;13:1690716. doi: 10.3389/fcvm.2026.1690716 (PMC13083164; doi:10.3389/fcvm.2026.1690716)
Supplement: Supplementary file 4 [file Supplementaryfile1.docx]

**Table S1. Representative Examples of Preprocessed Clinical Data Used as LLM Inputs**

| **No** | **sex** | **age** | **date of surgery** | **CTA** | **TTE** | **ECG** | **CAG** |
| --- | --- | --- | --- | --- | --- | --- | --- |
| 0 | 0 | 74 | 2022.11.7 | Access: Transthoracic Image Quality: Grade C Items: 2D, M-mode, Color Doppler (Pulse Wave, Continuous Wave), Tissue Doppler I. Main M-mode Measurements (Unit: mm):Aortic Root Diameter 33 Left Atrial Diameter 33 LVEDD (Left Ventricular End-Diastolic Diameter) 43 LVESD (Left Ventricular End-Systolic Diameter) 27 Interventricular Septal Thickness 9 Left Ventricular Posterior Wall Thickness 8 II. 2D Echocardiogram Description:No significant enlargement of heart chambers No thickening of the LV wall; no significant abnormalities in regional wall motion of the LV segments observed in the resting state Localized thickening and increased echogenicity of the aortic valve; opening is not restricted.No significant thickening of the remaining cardiac valves; opening is not restricted. III. Color Doppler Ultrasound Description: No significant shunting at the atrial or ventricular septal levels Mild mitral regurgitation. Trans-mitral diastolic flow: E=79cm/s, A=109cm/s, E/A=0.7.Trace aortic regurgitation Mild tricuspid regurgitation, maximum regurgitation velocity approx. 2.6m/s, estimated pulmonary artery systolic pressure (PASP) approx. 38mmHg. IV. Left Ventricular Function Measurement:LVEDV (ml) LVESV (ml) 26 LVEF (%) 69 LVFS (%) 39 Stroke Volume (ml) 59 V. Tissue Doppler Imaging:At the level of the mitral annulus: Septal E'=7.3cm/s, E/E'=10.8. LV lateral wall E'=8.4cm/s, E/E'=9.4. | Access: Transthoracic Image Quality: Grade C Items: 2D, M-mode, Color Doppler (Pulse Wave, Continuous Wave), Tissue Doppler I. Main M-mode Measurements (Unit: mm): Aortic Root Diameter: 33 Left Atrial Diameter: 33 LVEDD (Left Ventricular End-Diastolic Diameter): 43 LVESD (Left Ventricular End-Systolic Diameter): 27 Interventricular Septal Thickness: 9 LV Posterior Wall Thickness: 8 II. 2D Echocardiogram Description: No significant enlargement of heart chambers. No thickening of the LV wall; no significant abnormalities in regional wall motion observed in the resting state. Localized thickening and increased echogenicity of the aortic valve; opening is not restricted. No significant thickening of other cardiac valves; opening is not restricted. III. Color Doppler Ultrasound Description: No significant shunting at the atrial or ventricular septal levels. Mild mitral regurgitation. Trans-mitral diastolic flow: E=79cm/s, A=109cm/s, E/A=0.7. Trace aortic regurgitation. Mild tricuspid regurgitation, maximum regurgitation velocity approx. 2.6m/s, estimated pulmonary artery systolic pressure (PASP) approx. 38mmHg. IV. LV Function Measurement: LVEDV (ml): 85 LVESV (ml): 26 LVEF (%): 69 LVFS (%): 39 Stroke Volume (ml): 59 V. Tissue Doppler Imaging: Mitral annulus level: Septal E'=7.3cm/s, E/E'=10.8. LV lateral wall E'=8.4cm/s, E/E'=9.4. | Heart Rate: 57 bpm  P-R: 168 ms  QRS: 80 ms  QRS Axis: +26°  QT/QTc: 428/417 ms | The patient was placed in a supine position.  Conventional disinfection and draping were performed, and 2% lidocaine was used for local anesthesia of the right radial artery.  After successful puncture, a vascular sheath was inserted. Angiography showed:  RCA: 30% stenosis in the mid-segment and 20% stenosis in the distal segment; LM: 20% stenosis in the distal segment;  LAD: 85% stenosis at the ostium and myocardial bridging in the mid-segment; LCX: No significant stenosis observed. |
| 1 | 1 | 50 | 2024.7.5 | Access: Transthoracic: Items: 2D, M-mode, Color Doppler (Pulse Wave, Continuous Wave) Cardiac M-mode and 2D Measurements: (Unit: mm) M-mode LV Function Measurement Aortic Root Diameter: 30 LVEDV: 99 Left Atrial Diameter: 36 LVESV: 35 LVEDD: 46 LVEF (%): 65 LVESD: 30 LVFS (%): 35 Interventricular Septal Thickness: 9 Left Ventricular Posterior Wall Thickness: 9 I. 2D Ultrasound Description: Internal diameters of heart chambers are within normal range. Relationship and diameters of the great vessels are normal. Normal thickness of the interventricular septum and LV/RV walls; no significant abnormalities in regional wall motion of the LV segments observed in the resting state.No significant abnormalities in the morphology, structure, or opening/closing motion of all valves. No significant abnormalities in the pericardial cavity. Aneurysmal bulging in the mid-atrial septum towards the right atrium, measuring 21x10 mm. II. Color Doppler Ultrasound Description: No significant shunting at the atrial or ventricular septal levels. Trace mitral regurgitation observed. No significant aortic regurgitation. Trace tricuspid regurgitation observed. Trace pulmonary regurgitation observed. Trans-mitral diastolic flow: E=67cm/s, A=85cm/s, E/A=0.78. III. Tissue Doppler Imaging: At the level of the mitral annulus: Septal E'=9cm/s, E/E'=7. | Access: Transthoracic: Items: 2D, M-mode, Color Doppler (Pulse Wave, Continuous Wave) Cardiac M-mode and 2D Measurements: (Unit: mm) Aortic Root Diameter: 30 Left Atrial Diameter: 36 LVEDD (Left Ventricular End-Diastolic Diameter): 46 LVESD (Left Ventricular End-Systolic Diameter): 30 Interventricular Septal Thickness: 9 LV Posterior Wall Thickness: 9 M-mode LV Function Measurement LVEDV: 99 LVESV: 35 LVEF (%): 65 LVFS (%): 35 I. 2D Ultrasound Description: Internal diameters of all heart chambers are within normal range. Great vessel relationship and diameters are normal. Normal thickness of the interventricular septum and LV/RV walls; no significant abnormalities in regional wall motion observed in the resting state. No significant abnormalities in morphology, structure, or opening/closing motion of all valves. No significant abnormalities in the pericardial cavity. Aneurysmal bulging in the mid-atrial septum towards the right atrium, measuring 21x10 mm. II. Color Doppler Ultrasound Description: No significant shunting at the atrial or ventricular septal levels. Trace mitral regurgitation observed. No significant aortic regurgitation. Trace tricuspid regurgitation observed. Trace pulmonary regurgitation observed. Trans-mitral diastolic flow: E=67cm/s, A=85cm/s, E/A=0.78. III. Tissue Doppler Imaging: Mitral annulus level: Septal E'=9cm/s, E/E'=7. | Heart Rate: 62 bpm  P-R: 146 ms  QRS: 104 ms  QRS Axis: +70°  QT/QTc: 392/395 ms | The patient was placed supine in the catheterization lab for coronary angiography.  Routine disinfection and draping of the forearm and hand were performed.  Following local anesthesia with 2% lidocaine,  a 6F arterial sheath was inserted via right radial artery puncture.  Selective left and right coronary angiography was performed using TIG, JL3.5, and JR3.5 catheters,  which showed: LM and RCA: Normal visualization with no stenosis; LAD: 90% stenosis in the mid-segment;  LCX: 20% stenosis in the proximal segment and 30% stenosis in the distal segment; Right-dominant coronary artery system. |

**Table S2. Comprehensive Performance Metrics of Ensemble Strategies From Nested Cross-Validation**

| **method** | **metric** | **mean** | **std** | **ci95_low** | **ci95_high** |
| --- | --- | --- | --- | --- | --- |
| Best single | f1 | 0.8072 | 0.0198 | 0.7586 | 0.8387 |
| Best single | accuracy | 0.6821 | 0.0308 | 0.6111 | 0.7368 |
| Best single | roc_auc | 0.933 | 0.0665 | 0.7821 | 1 |
| Best single | brier | 0.1551 | 0.0239 | 0.1159 | 0.2099 |
| Best single | ece | 0.2102 | 0.0285 | 0.152 | 0.2749 |
| Best single | mce | 0.6616 | 0.1108 | 0.4667 | 0.8889 |
| Best single | sensitivity | 0.9838 | 0.0376 | 0.8462 | 1 |
| Best single | specificity | 0.0492 | 0.0873 | 0 | 0.3333 |
| Best single | ppv | 0.6851 | 0.0214 | 0.6471 | 0.7333 |
| Best single | npv | 0.2148 | 0.3798 | 0 | 1 |
| standard | f1 | 0.7945 | 0.0529 | 0.6667 | 0.88 |
| standard | accuracy | 0.7029 | 0.0656 | 0.5556 | 0.8333 |
| standard | roc_auc | 0.9613 | 0.0363 | 0.8718 | 1 |
| standard | brier | 0.1359 | 0.0171 | 0.1025 | 0.1716 |
| standard | ece | 0.2114 | 0.0308 | 0.1518 | 0.2691 |
| standard | mce | 0.5809 | 0.1106 | 0.3258 | 0.7926 |
| standard | sensitivity | 0.8595 | 0.1157 | 0.6154 | 1 |
| standard | specificity | 0.3743 | 0.2432 | 0 | 0.8333 |
| standard | ppv | 0.7508 | 0.0659 | 0.6667 | 0.9 |
| standard | npv | 0.5013 | 0.2849 | 0 | 1 |
| Adv global | f1 | 0.88 | 0.0642 | 0.75 | 1 |
| Adv global | accuracy | 0.8347 | 0.0857 | 0.6667 | 1 |
| Adv global | roc_auc | 0.8977 | 0.0803 | 0.7051 | 1 |
| Adv global | brier | 0.1216 | 0.0571 | 0.0205 | 0.243 |
| Adv global | ece | 0.1303 | 0.062 | 0.0367 | 0.2676 |
| Adv global | mce | 0.4596 | 0.209 | 0.125 | 0.8585 |
| Adv global | sensitivity | 0.9001 | 0.0978 | 0.6667 | 1 |
| Adv global | specificity | 0.6973 | 0.2237 | 0.1667 | 1 |
| Adv global | ppv | 0.8712 | 0.0852 | 0.7143 | 1 |
| Adv global | npv | 0.7972 | 0.1762 | 0.5 | 1 |
| Adv group | f1 | 0.9207 | 0.0558 | 0.7826 | 1 |
| Adv group | accuracy | 0.8926 | 0.075 | 0.7222 | 1 |
| Adv group | roc_auc | 0.9567 | 0.0486 | 0.8332 | 1 |
| Adv group | brier | 0.0777 | 0.0398 | 0.0165 | 0.1728 |
| Adv group | ece | 0.139 | 0.0415 | 0.0669 | 0.2303 |
| Adv group | mce | 0.5315 | 0.1758 | 0.2306 | 0.8921 |
| Adv group | sensitivity | 0.9249 | 0.081 | 0.75 | 1 |
| Adv group | specificity | 0.8248 | 0.179 | 0.3333 | 1 |
| Adv group | ppv | 0.9235 | 0.0721 | 0.75 | 1 |
| Adv group | npv | 0.8579 | 0.142 | 0.5714 | 1 |

**Table S3. PCI Recommendation Rates, Sensitivity, and Specificity of Individual LLMs**

| **Model** | **PCI Recommendation Rate Aggressiveness** | **Sensitivity** | **Specificity** |
| --- | --- | --- | --- |
| Llama-3.3-70B-Instruct | 0.9654 | 0.9947 | 0.0963 |
| Baichuan4-Turbo | 0.9343 | 0.9929 | 0.1889 |
| Llama-3.1-405B | 0.92 | 0.9647 | 0.1741 |
| o3-Mini | 0.8984 | 0.9647 | 0.2407 |
| Gemini-2.0-Flash-Thinking | 0.8805 | 0.9753 | 0.3185 |
| Claude-3.5-Sonnet | 0.8674 | 0.9718 | 0.3519 |
| DeepSeek-V3 | 0.8483 | 0.9559 | 0.3778 |
| Claude-3.7-Sonnet | 0.8339 | 0.9453 | 0.4 |
| Grok-2 | 0.8292 | 0.9365 | 0.3963 |
| Qwen-Max | 0.8112 | 0.903 | 0.3815 |
| Gemini-2.0-Pro | 0.6882 | 0.7866 | 0.5185 |
| DeepSeek-R1 | 0.6332 | 0.7513 | 0.6148 |
| Doubao-1.5-Pro-32K | 0.4659 | 0.5855 | 0.7852 |
| Grok-3 | 0.4349 | 0.5309 | 0.7667 |
| Doubao-1.5-Pro-256K | 0.4265 | 0.5273 | 0.7852 |
